# A role for the Cockayne Syndrome B (CSB)-Elongin ubiquitin ligase complex in signal-dependent RNA polymerase II transcription

**DOI:** 10.1016/j.jbc.2021.100862

**Published:** 2021-06-09

**Authors:** Juston C. Weems, Brian D. Slaughter, Jay R. Unruh, Kyle J. Weaver, Brandon D. Miller, Kym M. Delventhal, Joan W. Conaway, Ronald C. Conaway

**Affiliations:** 1 Stowers Institute for Medical Research, Kansas City, Missouri, USA; 2 Department of Biochemistry & Molecular Biology, University of Kansas Medical Center, Kansas City, Kansas, USA

**Keywords:** transcription regulation, RNA polymerase II, transcription elongation factor, ubiquitin ligase, glucocorticoid receptor, fluorescence resonance energy transfer (FRET), chromatin immunoprecipitation (ChIP), Elongin, Cockayne syndrome B (CSB), AP-FRET, acceptor photobleaching–fluorescence resonance energy transfer, ChIP, chromatin immunoprecipitation, CSB, Cockayne syndrome B, DTT, dithiothreitol, ELOA, Elongin A, ER, endoplasmic reticulum, GB, gene body, GR, glucocorticoid receptor, RNAPII, RNA polymerase II

## Abstract

The Elongin complex was originally identified as an RNA polymerase II (RNAPII) elongation factor and subsequently as the substrate recognition component of a Cullin-RING E3 ubiquitin ligase. More recent evidence indicates that the Elongin ubiquitin ligase assembles with the Cockayne syndrome B helicase (CSB) in response to DNA damage and can target stalled polymerases for ubiquitylation and removal from the genome. In this report, we present evidence that the CSB-Elongin ubiquitin ligase pathway has roles beyond the DNA damage response in the activation of RNAPII-mediated transcription. We observed that assembly of the CSB-Elongin ubiquitin ligase is induced not just by DNA damage, but also by a variety of signals that activate RNAPII-mediated transcription, including endoplasmic reticulum (ER) stress, amino acid starvation, retinoic acid, glucocorticoids, and doxycycline treatment of cells carrying several copies of a doxycycline-inducible reporter. Using glucocorticoid receptor (GR)-regulated genes as a model, we showed that glucocorticoid-induced transcription is accompanied by rapid recruitment of CSB and the Elongin ubiquitin ligase to target genes in a step that depends upon the presence of transcribing RNAPII on those genes. Consistent with the idea that the CSB-Elongin pathway plays a direct role in GR-regulated transcription, mouse cells lacking the Elongin subunit Elongin A exhibit delays in both RNAPII accumulation on and dismissal from target genes following glucocorticoid addition and withdrawal, respectively. Taken together, our findings bring to light a new role for the CSB-Elongin pathway in RNAPII-mediated transcription.

Elongin is a multifunctional RNA polymerase II (RNAPII) regulator that was first discovered as an RNAPII elongation factor capable of stimulating the overall rate of elongation through a direct interaction with transcribing RNAPII (1, 2). Elongin is a heterotrimer composed of a transcriptionally active A subunit and two smaller Elongin B and C subunits, which form a stable heterodimer that binds to a BC-box motif in Elongin A and upregulates its transcription activity (3). The Elongin complex was subsequently found to assemble with Cullin family member CUL5 and RING finger protein RBX2 to form an E3 ubiquitin ligase (4, 5) that plays a critical role in the DNA damage response by targeting RNAPII stalled at sites of DNA damage for ubiquitylation and removal from the genome (6, 7, 8, 9). Assembly of the Elongin ubiquitin ligase is a tightly regulated process rapidly induced by treatment of cells with a variety of DNA damaging agents or drugs that cause RNAPII stalling (10, 11).

Recently, we found that the Elongin ubiquitin ligase functions with the Cockayne syndrome B (CSB) protein during the DNA damage response (11). *ERCC6*, the gene encoding CSB, is mutated in Cockayne syndrome, a genetic disorder in which affected individuals exhibit neuronal, growth, and developmental abnormalities as well as extreme sun sensitivity (12). *ERCC6* mutations give rise to defects in multiple nuclear processes, including transcription and transcription-coupled DNA repair (13). During the DNA damage response, the Elongin ubiquitin ligase and CSB assemble into a higher order complex that is targeted to sites of DNA damage (11); whether this complex includes additional proteins remains to be determined. Although assembly of the Elongin ubiquitin ligase does not require CSB, stable recruitment of the Elongin ubiquitin ligase to sites of DNA damage is strongly CSB-dependent (11). In light of previous evidence that CSB can act as a sensor of stalled RNAPII (13, 14, 15, 16, 17), it is possible that the CSB-Elongin ubiquitin ligase complex is targeted directly to stalled polymerases by CSB.

Early on in our investigation of the Elongin ubiquitin ligase, we discovered that its assembly is potently induced not only by DNA damage and drugs that cause RNAPII stalling, but also by other stimuli such as ER stress, amino acid starvation, and hormone treatment of cells (10). These findings raised the intriguing possibility that the Elongin ubiquitin ligase, with or without CSB, might have a role in processes besides the DNA damage response. Since a shared feature of ER stress, amino acid starvation, and hormone treatment is that they all result in activation of robust RNAPII transcription programs, we considered the possibility that the CSB-Elongin ubiquitin ligase complex might have roles not only in DNA repair, but also in RNAPII transcription *per se*.

In this report, we present evidence consistent with this model. We show that, like assembly of the Elongin ubiquitin ligase, assembly of CSB with Elongin and the ubiquitin ligase component CUL5 is induced by ER stress, amino acid starvation, and retinoic acid and glucocorticoids. Furthermore, we show that assembly of CSB with Elongin and CUL5 can also be induced simply by doxycycline treatment of U2OS cells carrying chromosomally integrated copies of a doxycycline-inducible reporter gene, arguing that activation of a simple RNAPII transcription program is sufficient to serve as a signal for induction of the CSB-Elongin pathway. Finally, we present evidence (i) that CSB and components of the Elongin ubiquitin ligase are rapidly recruited to glucocorticoid-activated genes in a step that requires the presence of a transcribing polymerase on the gene and (ii) that cells lacking Elongin A exhibit a modest delay in accumulation of RNAPII upon induction of glucocorticoid-responsive genes and a substantial delay in removal of RNAPII from these genes following removal of glucocorticoids. Taken together, our findings bring to light a role for the CSB-Elongin pathway in the regulation of RNAPII transcription.

## Results and discussion

### Multiple signaling pathways drive assembly of the CSB-Elongin ubiquitin ligase complex

To begin to explore the possibility that CSB and Elongin might function together outside the DNA damage response, we sought to determine whether interaction of the Elongin ubiquitin ligase with CSB is increased by signals other than DNA damage. Because we previously observed that assembly of the Elongin ubiquitin ligase can be induced by thapsigargin, which induces ER stress, histidinol, which mimics amino acid starvation, and the steroid hormone retinoic acid (10), we asked whether these same agents increase binding of the Elongin ubiquitin ligase to CSB. To accomplish this, we assessed the interactions among CSB, Elongin A (ELOA), and CUL5 in individual living cells using an acceptor photobleaching–fluorescence resonance energy transfer (AP-FRET) assay we previously used to characterize assembly of the Elongin ubiquitin ligase and its interaction with CSB in response to DNA damage (10, 11). During FRET, energy is transferred by a donor fluorophore to an acceptor fluorophore. Upon energy transfer, which typically occurs only if the distance between donor and acceptor is ∼100 Å or less, the emission of the donor is quenched and that of the acceptor is enhanced. In these assays, FRET efficiency is measured by comparing donor emission before photobleaching to donor emission after acceptor photobleaching, when the acceptor is no longer available to absorb energy from the donor. In an AP-FRET experiment, the protein labeled with acceptor fluorophore is expressed at levels substantially higher than the protein with the donor fluorophore, so FRET is not limited by the availability of acceptor (18). In addition, we routinely confirm that AP-FRET signals are independent of levels of donor expression (Fig. S1).

**Figure 1:**
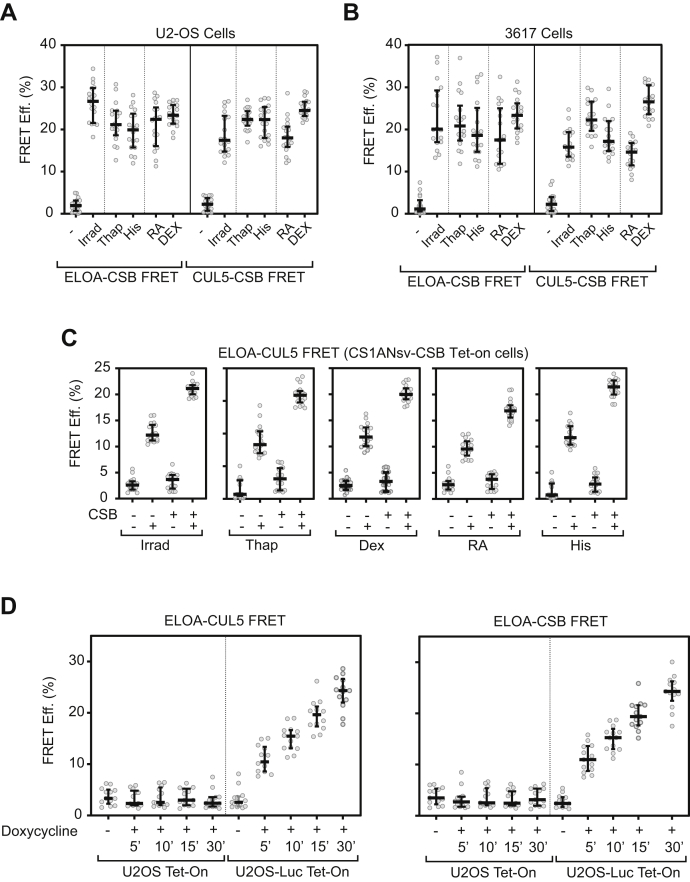
**Increased assembly of the CSB-Elongin ubiquitin ligase complex in response to signals that activate transcription.** AP-FRET efficiency (FRET Eff.) was measured in U2OS cells (*panel A*), 3617 cells (*panel B*), CS1ANsv-CSB Tet-on cells (*panel C*), and U2OS Tet-On or U2OS-Luc Tet-On (*panel D*) expressing either R110Direct-labeled Halo-ELOA and GFP-CSB or TMRDirect-labeled Halo-ELOA and mCherry CUL5 and subjected to the treatments indicated in the figure. DEX, dexamethasone; His, histidinol; Irrad, laser microirradiation; RA, retinoic acid; Thap, thapsigargin.

In the experiments described here, GFP-CSB and Halo-ElOA or GFP-CSB and mCherry-CUL5 were transiently expressed in human U2OS osteosarcoma cells (Fig. 1*A*) or a mouse cell line, 3617, which harbors ∼200 tandemly repeated copies of an MMTV-LTR-driven, glucocorticoid-inducible model gene (19) (Fig. 1*B*). We then measured total nuclear AP-FRET before and after stimulus, using laser-induced DNA damage as a positive control. Consistent with our previous results (10, 11), we observed low but measurable AP-FRET between Halo-ELOA and GFP-CSB and between mCherry-CUL5 and GFP-CSB in unstimulated cells, indicating that there is a baseline level of interaction between these proteins at steady state. In cells treated with thapsigargin, histidinol, retinoic acid, or the synthetic glucorticoid dexamethasone, AP-FRET signals between ELOA and CSB and between CUL5 and CSB were substantially increased over the low AP-FRET signals seen in untreated cells. This finding suggests that, like DNA damage, these treatments cause an increase in levels of the higher order CSB-Elongin ubiquitin ligase complex in cells.

### Signal-induced Elongin ubiquitin ligase assembly does not require CSB

We previously observed that CSB is not essential for, but modestly enhances, assembly of the Elongin ubiquitin ligase during the DNA damage response (11). We therefore asked whether CSB is needed for assembly of the Elongin ubiquitin ligase in response to other signaling pathways. To do so, we took advantage of a derivative of human CS1Ansv cells, which were derived from a Cockayne Syndrome patient and lack a functional CSB gene (20, 21). This cell line, designated CS1ANsv-CSB Tet-on, has been engineered to inducibly express wild-type CSB and has been used to study CSB-dependent processes (22). In our experiments, Halo-ELOA and mCherry-CUL5 were transiently expressed in CS1ANsv-CSB Tet-on cells in which CSB expression was induced or not, and AP-FRET between Halo-ELOA and mCherry-CUL5 was measured following treatment of cells with thapsigargin, histidinol, dexamethasone, or retinoic acid.

As shown in Figure 1*C*, induction of CSB expression was not essential for assembly of the Elongin ubiquitin ligase in response to any of these stimuli. As we observed previously (11), ELOA-CUL5 AP-FRET signals increased after UV irradiation when CSB expression was not induced, but increased roughly 2-fold more in cells expressing CSB. These findings are consistent with results of a previous study in which we observed that there was only a modest increase in the DNA damage-driven interaction between Elongin A and CUL5 upon induction of CSB expression in this cell line, even though recruitment of the Elongin ubiquitin ligase to sites of DNA damage was severely defective when CSB expression was not induced (11). Similarly, ELOA-CUL5 AP-FRET signals increased after treatment with thapsigargin, histidinol, dexamethasone, or retinoic acid in cells lacking CSB, but again were increased about approximately 2-fold more when CSB expression was induced with doxycycline. These observations further reinforce the idea that CSB is likely not essential for assembly of the Elongin ubiquitin ligase, but does enhance or stabilize its assembly. Thus, assembly of the CSB-Elongin ubiquitin ligase complex appears to proceed similarly in response to DNA damage and to ER stress, amino acid starvation, and steroids, including retinoic acid and dexamethasone. Because these latter stimuli are not known to induce extensive DNA damage, our findings are consistent with the idea that the CSB-Elongin axis plays a role in cellular processes beyond the DNA damage response.

### Assembly of the CSB-Elongin ubiquitin ligase complex in response to transcription activation

Our evidence that DNA damage is not the sole inducer of assembly of the CSB-Elongin ubiquitin ligase complex led us to ask what signal(s) might be responsible for driving assembly. Although the DNA damage response, ER stress, amino acid starvation, and hormones can activate different cellular processes, they share one common endpoint: all lead to activation of robust transcription programs. In view of this shared feature and of the known roles of Elongin, the Elongin ubiquitin ligase, and CSB in regulation of transcribing RNAPII, we sought to explore the possibility that transcription activation *per se* might be sufficient to drive assembly of CSB with the Elongin ubiquitin ligase and activation of the CSB-Elongin axis.

As one way to explore this question, we took advantage of a pair of cell lines: U2OS-Luc Tet-On cells, which carry multiple copies of a chromosomally integrated, doxycycline-inducible luciferase reporter driven by an RNAPII promoter, and parental U2OS Tet-On cells, which lack the doxycycline-inducible luciferase reporter gene. By subjecting these cells to brief treatment with doxycycline, we could ask whether rapid activation of new transcription is sufficient to drive binding of CSB to Elongin A and CUL5.

U2OS-Luc Tet-On and parental cells without the doxycycline-inducible reporter, transiently expressing Halo-ELOA and either mCherry-CUL5 or mCherry CSB, were treated with 0.5 μg/ml of doxycycline for 5, 10, 15, or 30 min. Doxycycline treatment did not increase either ELOA-CUL5 or ELOA-CSB AP-FRET signals in parental U2OS Tet-On cells; however, AP-FRET signals began to increase rapidly after addition of doxycycline to induce transcription of the luciferase reporter in U2OS-Luc Tet-On cells (Fig. 1*D*). Taken together, these results support the conclusion that rapid activation of new transcription is sufficient to lead to substantial increases in assembly of the CSB-Elongin ubiquitin ligase complex. Furthermore, they point to RNAPII transcription as a signal for activation of the CSB-Elongin axis.

### CSB, Elongin, and CUL5 are recruited to actively transcribed glucocorticoid-regulated genes

Our evidence that activation of RNAPII transcription provides a signal leading to assembly of the CSB-Elongin ubiquitin ligase complex led us to ask whether CSB, Elongin, and the ubiquitin ligase component CUL5 are all recruited to newly activated genes. In initial experiments, we used live cell imaging to address this question in mouse 3617 cells, which have been used extensively in imaging studies of RNAPII transcription regulation (19, 23, 24, 25). In total, 3617 cells contain a chromosomally integrated, tandem array consisting of ∼200 copies of a glucocorticoid-activated, MMTV promoter-reporter gene cassette. In addition, these cells express glucocorticoid receptor fused to GFP (GR-GFP). In the presence of glucocorticoids, GR-GFP binds glucocorticoid-responsive elements in the MMTV promoter and accumulates at the array, where it can be visualized as fluorescent puncta.

**Figure 2:**
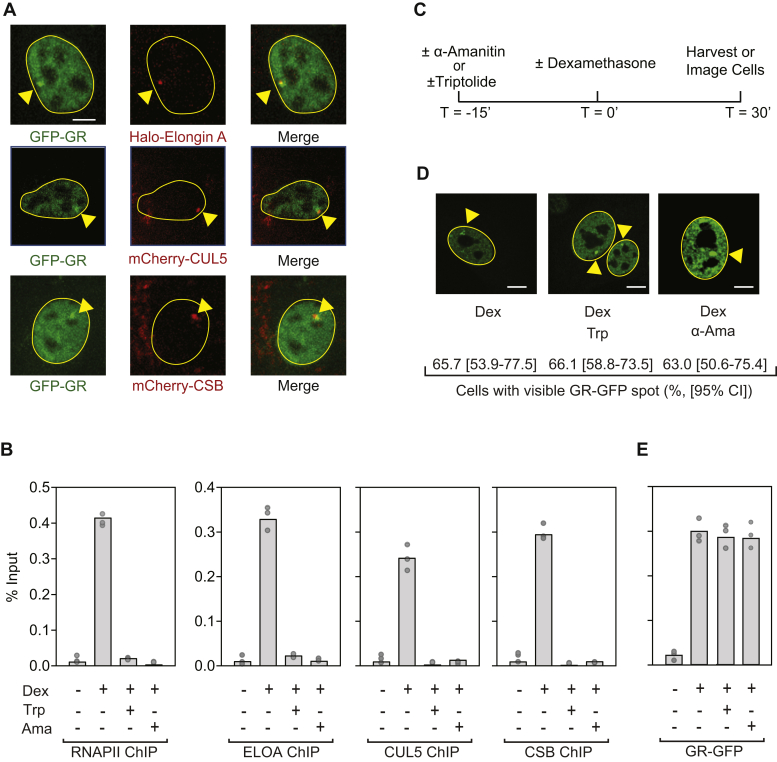
**Triptolide blocks GR-induced recruitment of CSB, Elongin A, and CUL5 to the MMTV LTR.***A*, representative images showing colocalization of GFP-GR, Elongin A, CUL5, and CSB at the MMTV array in dexamethasone-treated 3617 cells. *B*, ChIP with antibodies against the indicated proteins was performed with chromatin prepared from 3617 cells treated or not with 10 μM α-amanitin, 500 μM triptolide, and 100 nM dexamethasone according to the protocol shown in *panel C*. *D*, representative images of 3617 cells treated with dexamethasone, without or with triptolide or α-amanitin as diagrammed in *panel C*. The percentage of cells with visible GR-GFP spots was determined by counting at least 300 cells in each of three independent experiments and is expressed as the mean value, with upper and lower limits of the 95% confidence interval. *Panel E*, ChIP with anti-GFP antibodies, performed with chromatin from 3617 cells treated or not with 10 μM α-amanitin, 500 μM triptolide, and 100 nM dexamethasone. Scale bars = 9 μm. Ama, α-amanitin; CI, confidence interval; Dex, dexamethasone; Trp, triptolide.

In total, 3617 cells were transiently transfected with plasmids encoding Halo-ELOA, mCherry-CUL5, or mCherry-CSB and treated with dexamethasone. As shown in Figure 2*A*, puncta of Halo-Elongin A, mCherry-CUL5, and mCherry-CSB were all detected along with GFP-GR at the MMTV array in dexamethasone-treated cells. Consistent with these observations, in chromatin immunoprecipitation (ChIP) experiments, we observed that endogenous Elongin A, CUL5, and CSB are recruited with RNAPII to the MMTV promoter region following dexamethasone treatment of cells (Fig. 2*B*). Taken together, these observations are consistent with the idea that the CSB-Elongin ubiquitin ligase complex is recruited to the MMTV array coincident with activation of RNAPII transcription.

CSB, Elongin, and the Elongin ubiquitin ligase are all thought to recognize and bind specifically to transcribing RNAPII to execute their functions in RNAPII transcription (2, 8, 9, 14, 26). We therefore sought to determine whether the presence of actively transcribing RNAPII on GR-activated genes might signal recruitment of CSB and the Elongin ubiquitin ligase complex. To begin to address this question, we used the protocol outlined in Figure 2*C*, which takes advantage of the RNAPII transcription inhibitors triptolide or α-amanitin. Triptolide blocks RNAPII initiation by inhibiting promoter opening dependent on TFIIH XPB ATPase/translocase activity (27), whereas α-amanitin interferes with transcription by inhibiting nucleotide addition to nascent RNA chains (28, 29, 30). In these experiments, cells were treated for 15 min with vehicle (DMSO) or with one of the RNAPII inhibitors prior to induction of GR-activated transcription with dexamethasone. If active transcription is needed to bring the CSB-Elongin complex to GR-activated genes, both triptolide and α-amanitin would be expected to block CSB-Elongin recruitment.

The results of ChIP experiments indicate that dexamethasone-induced accumulation of RNAPII, as well as ELOA, CUL5, and CSB near the MMTV reporter gene transcription start sites in the array was blocked by both inhibitors (Fig. 2*B*). In contrast, neither inhibitor affected dexamethasone-induced targeting of GR to the MMTV array. As shown in Figure 2*D*, neither triptolide nor α-amanitin prevented formation of GR-GFP puncta at the MMTV array in dexamethasone-treated cells, and GR-GFP recruitment to MMTV promoter regions in the array was unaffected by pretreatment with either inhibitor as measured by ChIP (Fig. 2*E*). Taken together, these results are consistent with the idea that RNAPII transcription is needed for recruitment of the CSB-Elongin A ubiquitin ligase to MMTV reporter genes in the MMTV array.

**Figure 3:**
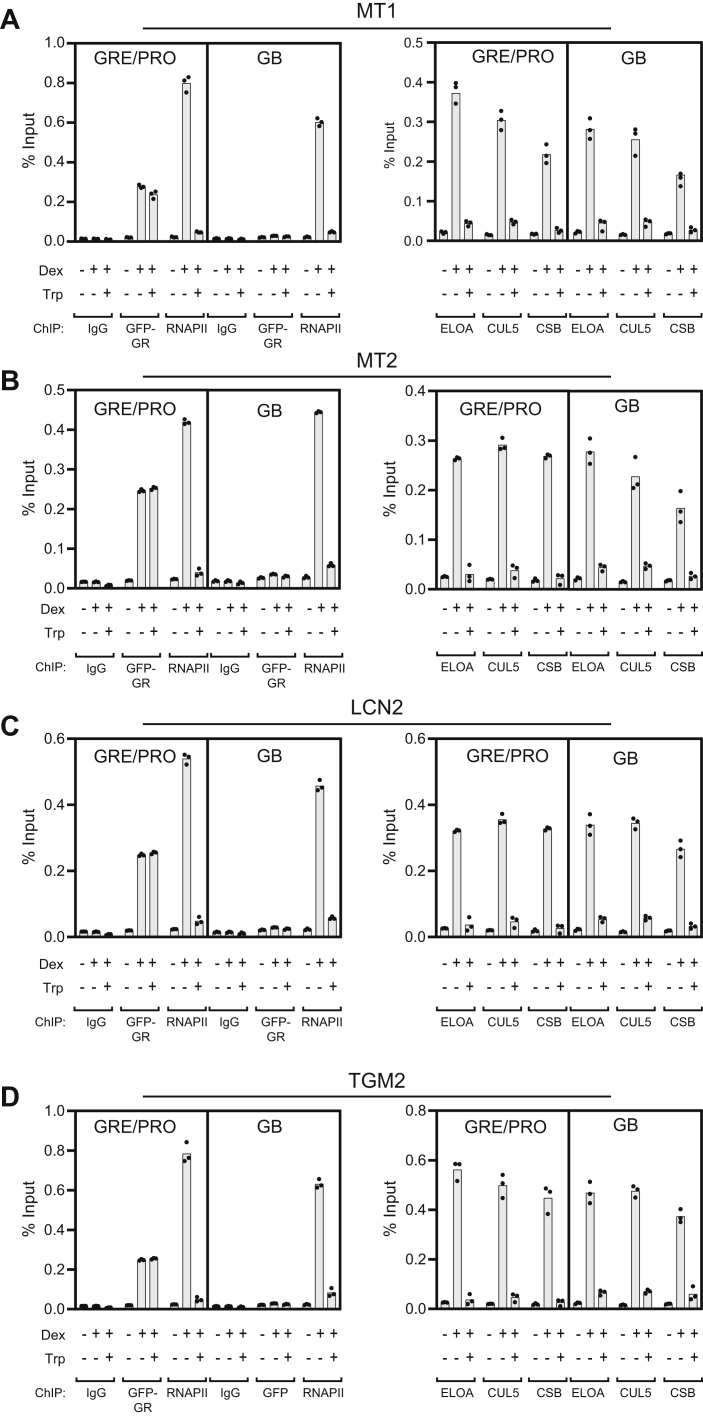
**Triptolide blocks recruitment of CSB, Elongin A, and CUL5 to endogenous glucocorticoid-responsive genes.** In total, 3617 cells were treated for 5 min with vehicle (DMSO) or 500 μM triptolide (Trp) to block new transcription initiation before addition of DMSO or 100 nM dexamethasone (Dex). Twenty minutes later, cells were cross-linked, and ChIP was performed using antibodies against the indicated proteins. Primer sets (Table S1) corresponding to regions near the GR response elements and promoter (GRE/PRO) or near the 3′ end of each gene (GB) were used to amplify immunoprecipitated DNA. These genes fall into two classes: “pre-programmed genes” (LCN2 and MT2), where GR-binding sites are associated with DNase I hypersensitive sites that exist prior to exposure to GR ligand, and “de novo genes” (MT1 and TGM2) where DNase I hypersensitive sites are induced by ligand (25).

Activation of the MMTV reporter genes in the array represents an unusual circumstance where potentially hundreds of transcripts in a relatively small region are activated simultaneously. To determine whether endogenous genes behave similarly to genes in the MMTV array, we performed ChIP experiments at the endogenous GR-activated genes *MT1*, *MT2*, *LCN2*, and *TGM2*. In these experiments, we measured GFP-GR, RNAPII, ELOA, CUL5, and CSB occupancy at gene 5′ ends, near GRE and promoter regions (GRE/PRO), and at gene body (GB) sites near the 3′ end of each transcript (Fig. 3 and Table S1).

In all four genes, we observed a very similar pattern. RNAPII occupancy was strongly induced at gene 5′ ends and within the gene body after dexamethasone treatment alone; however, when triptolide was added to cells shortly before addition of dexamethasone, we observed little to no accumulation of RNAPII at either position, indicating that transcription activation of these glucocorticoid-responsive genes is effectively blocked by triptolide. As seen at the MMTV array, triptolide inhibition of RNAPII accumulation was not due to an inability of GFP-GR to accumulate at the promoters of these genes, since GR occupancy was indistinguishable in the presence and absence of triptolide. Similar to RNAPII, endogenous ELOA, CUL5, and CSB all accumulated at both the promoter regions and gene bodies of the *MT1*, *MT2*, *LCN2*, and *TGM2* genes in response to dexamethasone treatment, and triptolide blocked this effect. Taken together, these data support the model that the CSB-Elongin ubiquitin ligase complex is recruited to glucocorticoid-responsive genes in a transcription-dependent manner.

### Altered RNAPII dynamics at GR-regulated genes in cells lacking ELOA

In mammals, glucocorticoid hormones are secreted from the adrenal glands in an ultradian, or pulsatile, pattern with a periodicity of about 1 h (31, 32, 33, 34). When the natural GR ligand corticosterone is applied to cultured cells in a pulsatile manner, GR binds rapidly to its binding sites in promoters upon addition of corticosterone and is rapidly dismissed when ligand is removed. Similarly, at GR-regulated genes, both Pol II occupancy and transcription rapidly increase and decrease upon ligand addition and removal (24). In contrast, GR continues to occupy promoters for a considerable period of time when the same experiments are performed using synthetic GR ligands such as dexamethasone, perhaps because slow disassociation of the high-affinity dexamethasone ligand from GR prevents the rapid response to decreasing ligand levels seen with corticosterone (24).

**Figure 4:**
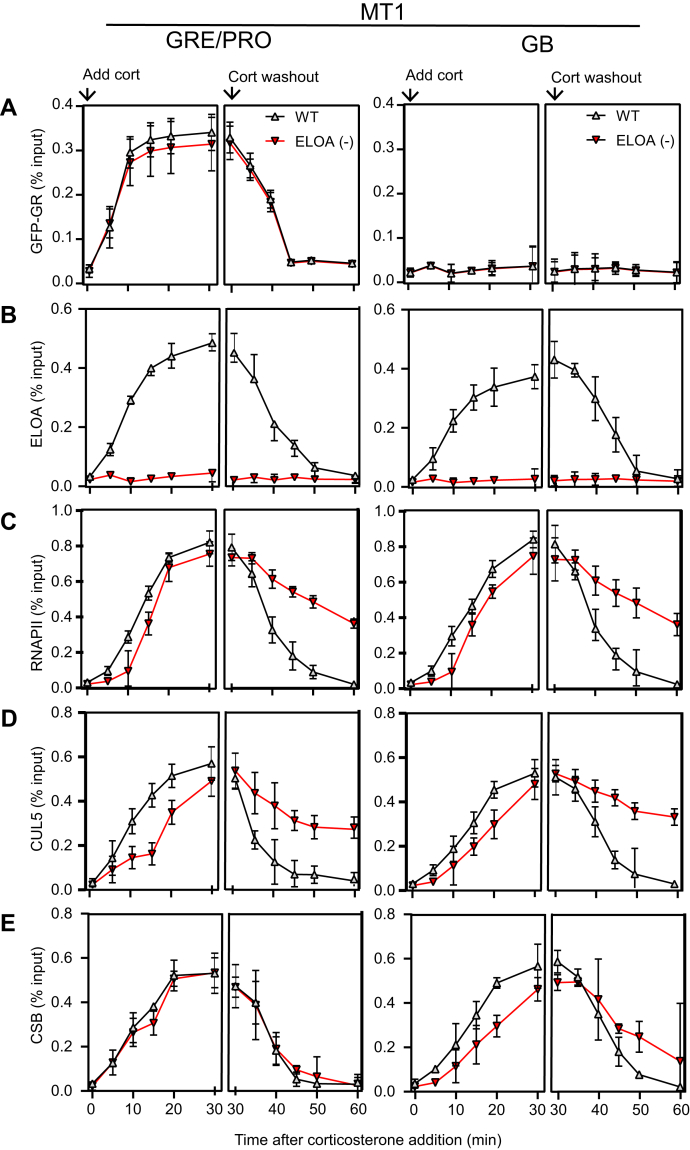
**Altered RNAPII, CUL5, and CSB dynamics at GR-regulated genes in cells lacking ELOA.** Wild-type or ΔELOA 3617 cells were either incubated with 100 nM corticosterone for up to 30 min or, in corticosterone washout experiments, were incubated with corticosterone for 30 min and then washed to remove corticosterone and incubated with hormone-free medium for up to 30 min. Crosslinked chromatin from these cells was subjected to ChIP with antibodies against the indicated proteins. Cort, corticosterone; GB, gene body; GRE/PRO, glucocorticoid response element and promoter region.

To explore potential role(s) of the CSB-Elongin pathway in GR-responsive transcription, we asked whether mutation of Elongin A affects either corticosterone-dependent activation of transcription, assessed by RNAPII ChIP near promoters and within gene bodies, or the rapid shut-off of transcription following corticosterone removal. We used CRISPR-Cas9 technology to generate 3617 ΔELOA cells, which are derived from mouse 3617 cells and do not express Elongin A. Confirming the absence of ELOA in 3617 ΔELOA cells, ELOA was not detected by immunoblotting in cell lysates (Fig. S2), and we detected no ELOA accumulation at either the endogenous *MT1* and *MT2* genes or the MMTV array after addition of corticosterone to these cells(Figs. 4 and S3).

Consistent with previous studies, we observed a rapid increase in GFP-GR occupancy at the *MT1*, *MT2*, and MMTV promoter regions, but not within gene bodies, after corticosterone addition. The rate and extent of increase GFP-GR occupancy in wild-type and ELOAΔ 3617 cells were indistinguishable, arguing that ELOA has no effect on corticosterone-responsive targeting of GR to these promoters. Following washout of corticosterone, GFP-GR occupancy at the MT1, MT2, and MMTV promoters was reduced to background levels within 15 min, and the decay of GFP-GR ChIP signals after washout in wild-type and mutant cells was indistinguishable (Figs. 4*A* and S3).

In contrast, the kinetics of RNAPII accumulation and dismissal were altered in 3617 ΔELOA cells. As shown in Figures 4*C* and S3, after addition of corticosterone, RNAPII accumulated rapidly at promoters and within gene bodies in wild-type cells. In ELOA-deficient cells, RNAPII accumulation was somewhat delayed, showing a reduction relative to wild type in the first 5–15 min, but reaching similar levels of occupancy by 20 min after corticosterone addition. The most striking differences, however, were observed after corticosterone removal. In wild-type cells, loss of RNAPII from promoter regions and gene bodies roughly paralleled loss of promoter-bound GFP-GR, consistent with prior evidence that transcription of GR-dependent genes is rapidly shut off following removal of corticosterone and loss of GR from promoters (24). However, in 3617 ΔELOA cells, we observed a substantial delay in loss of RNAPII from both promoters and gene bodies, arguing that ELOA, and potentially the entire Elongin ubiquitin ligase complex, contributes to rapid dismissal of RNAPII from these genes during transcriptional shutdown.

We next examined the effects of corticosterone addition and withdrawal on the kinetics of accumulation and loss of ELOA and CUL5 at these GR-regulated genes. In wild-type 3617 cells, the rates of ELOA accumulation and loss from the promoter regions and gene bodies of all three genes paralleled RNAP II occupancy (Figs. 4*B* and S3), consistent with evidence that ELOA functions through a direct interaction with transcribing RNAPII (2). Notably, CUL5 accumulation and loss closely follow those of RNAPII in both wild-type and ELOA-deficient 3617 cells (Figs. 4*D* and S3), suggesting that CUL5 can be recruited to transcribing RNAPII by a mechanism independent of ELOA. Indeed, results of AP-FRET experiments indicate that binding of CSB to CUL5 is reduced but readily detectable in ELOA(−) cells (Fig. S4), suggesting that that CUL5-CSB interactions could contribute to CUL5 recruitment. We cannot, however, rule out the possibility that CUL5 can be brought to transcribed genes by mechanism(s) independent of the CSB-Elongin axis, since it is a component of multiple Elongin BC-containing Cullin-RING ligases (4, 35).

Finally, we tested the effects of corticosterone addition and withdrawal on the kinetics of CSB accumulation and loss in wild-type and ΔELOA 3617 cells (Figs. 4*E* and S1). In wild-type cells, rates of CSB accumulation after corticosterone addition and loss after corticosterone withdrawal paralleled those of RNAPII, ELOA, and CUL5 at both promoter regions and gene bodies. Similar to RNAPII and CUL5, at gene bodies, corticosterone-dependent accumulation of CSB and loss of CSB after corticosterone withdrawal were delayed in the ELOA-deficient cells. Different than RNAPII and CUL5, however, the kinetics of CSB accumulation and loss in promoter regions were unaffected by loss of ELOA, suggesting that different mechanisms may control CSB occupancy in the vicinity of promoters and in gene bodies. Evidence consistent with the idea that CSB occupancy in more 5′ and 3′ gene regions may be regulated differently was also obtained in a previous analysis of the occupancy of Rad26p, the yeast ortholog of CSB, at the *GAL1* gene after DNA damage in yeast cells grown under conditions that support active *GAL1* transcription (36). In this study, it was observed that after DNA damage, Rad26p occupancy in more 3′ portions of the GAL1 ORF decreased coincident with DNA-damage-induced loss of RNAPII, but remained fairly constant at a more 5′ portion of *GAL1* even though RNAPII occupancy decreased similarly at 5′ and 3′ portions of the gene.

### Summary and perspective

In this report, we provide evidence consistent with a role for the CSB-Elongin pathway in activation and shutdown of signal-responsive RNAPII transcription. The Elongin ubiquitin ligase was initially found to assemble with CSB and function in the DNA damage response, where it targets RNAPII stalled at DNA lesions for ubiquitylation and proteasomal removal from the genome to facilitate DNA repair and restore normal transcription. We previously found that assembly of CSB with the Elongin ubiquitin ligase complex is a tightly regulated process, rapidly induced by treatment of cells with a large variety of DNA-damaging agents (11). The main findings of the current report are as follows: First, we show that assembly of CSB with Elongin A and the ubiquitin ligase component CUL5 is induced not only by genotoxic stress, but also by a variety of additional signals, such as ER stress, amino acid deprivation, and hormones. Second, we present evidence that, under these conditions, the signal for activation of the CSB-Elongin pathway is RNAPII transcription itself, by showing that assembly of CSB with Elongin and CUL5 can be induced simply by doxycycline treatment of U2OS cells carrying a doxycycline-inducible reporter, arguing that activation of this simple Pol II transcription program is sufficient to serve as a signal for induction of the CSB-Elongin pathway. Third, we show that upon treatment of cells with glucocorticoids, CSB and components of the Elongin ubiquitin ligase are rapidly recruited to glucocorticoid-induced genes in a step that requires an actively transcribing RNAPII on those genes, a finding consistent with previous evidence that Rad26p recruitment in yeast depends on active transcription (36). And finally, we demonstrate that loss of Elongin leads to a modest but highly reproducible delay in the increase in RNAPII occupancy at GR-regulated genes upon corticosterone addition to cells and a more substantial delay in the dismissal of RNAPII when corticosterone is withdrawn.

We do not yet know exactly how the CSB-Elongin pathway might regulate rates of RNAPII recruitment to and dismissal from GR-regulated genes. We believe it is reasonable to speculate that the CSB-Elongin ubiquitin ligase could function in transcription similarly to its role in the DNA damage response, where it removes stalled RNAPII from genes. It is known that the activation of RNAPII transcription at many genes is accompanied by rapid bursts of RNAPII initiations occurring, in some cases, at intervals of less than 10 s (37, 38, 39). Under these conditions, RNAPII collisions can lead to stalling and arrest of the enzyme shortly after initiation, and stalled RNAPII on a gene can present an impenetrable obstacle to trailing polymerases, thereby shutting down further transcription (40). Thus, the CSB-Elongin ubiquitin ligase could safeguard inducible RNAPII transcription by ensuring expeditious removal of stalled RNAPII from genes. In addition, a very recent study revealed that Elongin A depletion leads to a substantial decrease in phosphorylation of the RNAPII CTD on serine 2, suggesting it can contribute to the maturation of RNAPII elongation complexes (41). Together, contributions of Elongin and the Elongin ubiquitin ligase in each of these processes could contribute to the modest delay in RNAPII accumulation on genes seen in mouse cells lacking Elongin A. Our observation that mouse cells lacking Elongin A exhibit a marked delay in removal of RNAPII from glucocorticoid-induced genes following withdrawal of hormone argues that the CSB-Elongin pathway makes an important contribution to rapid changes in transcription rates in response to changing levels of signaling molecules, perhaps *via* removal of RNAPII by ubiquitin-dependent proteolysis following RNAPII ubiquitylation by the Elongin ubiquitin ligase. Alternatively, since Elongin stimulates the rate of transcript elongation *in vitro*, the delay in loss of RNAPII from genes in Elongin A-deficient cells following hormone withdrawal could, in principle, be due to decreased rates of elongation; however, two recent reports indicating that overall RNAPII elongation rates in cells lacking Elongin A are not dramatically changed argue against this model (41, 42). Finally, it is important to note that we do not yet know what fraction of Elongin is associated with CSB or CUL5 at any given point during activation and shut-off of glucocorticoid-regulated transcription. Hence, we cannot exclude the possibility that distinct subassemblies containing different combinations of Elongin, CSB, and/or ubiquitin ligase subunits function in different steps of transcription. Future experiments exploring the molecular basis of this phenotype should provide additional insight into molecular mechanism(s) by which the CSB-Elongin axis functions in signal-dependent RNAPII transcription.

## Experimental procedures

### Materials

HaloTag R110Direct Ligand G3221 and HaloTag TMRDirect Ligand G2991 were purchased from Promega. Hoechst solution (33,258; used at 1:1000 dilution), L-histidinol dihydrochloride (H6647; used at 2 mM), all-trans-Retinoic acid (R-2625; used at 10 μm), thapsigargin (T-9033; used at 300 nM), and triptolide (T-3652, used at 500 μM) were all from Sigma. α-Amanitin (used at 10 μM) was purchased from Sigma (A2263) or EMD/Millipore (129741). FuGENE HD transfection reagent was obtained from Promega.

### Cell lines and cell culture

U2OS cells (ATCC HTB-96) were cultured in phenol-red free McCoy's medium (Gibco, Invitrogen), at 37 °C in 5% CO_2_. U2OS Tet-on (RRID:CVCL_V335, ClonTech, gift of Mark Ginsberg, UC San Diego), U2OS-Luc Tet-on (RRID:CVCL_V336, ClonTech, gift of Michael Carey, UCLA), and CS1ANsv-CSB Tet-on cells (22) were cultured in phenol red free DMEM containing 10% Tet system-approved fetal bovine serum (Clontech) at 37 °C with 5% CO_2_ without antibiotics. Where indicated, cells were treated with 1.0 μg/ml doxycycline to induce either the Tet-on system or CSB expression 24 h prior to use for transfection. After transfection, phenol red free DMEM and McCoy's media were supplemented with 5% Glutamax, 10% charcoal stripped One Shot Fetal Bovine Serum (Gibco, Life Technologies), 100 U/ml of penicillin, and 100 μg/ml of streptomycin (Gibco, Life Technologies).

Mouse cell line 3617 has been previously described (Muller *et al*, J Cell Biol, 2001). In total, 3617 cells stably express GFP-tagged GR under the control of a tetracycline-off system (Walker *et al*., 1999) and contain at least 200 repeats of a 9-kb element composed of the MMTV promoter followed by ras and BPV genes (Kramer *et al*., 1999). In total, 3617 cells and derivatives were grown in phenol-red free DMEM supplemented 5% Glutamax and 10% charcoal-stripped One Shot Fetal Bovine Serum (Gibco, Life Technologies). The cells were maintained in the presence of 5 μg/ml tetracycline to suppress GFP-GR expression. A *TCEB3* null, 3617 derivative, designated 3617 ΔELOA, was generated using CRISPR-Cas9 technology as described (43). The guideRNA target site was selected as described and by evaluating the predicted on-target score and off-target potential (43). The guideRNA target sequence was synthesized as two complementary oligos 5′-ACCGACGCGAGCCCAGTTCCGGCG-3′ and 5′-AAACCGCCGGAACTGGGCTCGCGTC-3′ (Integrated DNA Technologies). The oligos contained 4 bp overhangs for direct cloning into pSpCas9(BB)-2A-GFP (PX458), a gift from Feng Zhang (Addgene plasmid # 48138; http://n2t.net/addgene:48138; RRID:Addgene_48138) (43). Genomic DNA was extracted from cell pellets from candidate mutant clones using a Promega Maxwell 16 instrument and Promega Maxwell 16 Tissue Kit. The genomic region around the guide RNA target was amplified by PCR and prepared for Sanger sequencing on an Applied Biosystems 3730 DNA Analyzer using ExoSap-IT Express (Thermo Fisher). The resulting sequence data was analyzed for mutations at the target site using TIDE: Tracking of Indels by Decomposition (44). Selected TCEB3 knockout cell lines were further analyzed by targeted deep-sequencing for on- and off-target edits. Cell pellets were lysed using QuickExtract DNA Extraction Solution (Lucigen). For each guideRNA used, the on-target location and one or two predicted off-target locations were amplified by PCR. A second round of PCR incorporated sample-specific dual barcodes. All amplicons were pooled, size-selected using SPRIselect beads (Beckman Coulter), and quantified on a Qubit fluorometer and evaluated on an Agilent Bioanalyzer to check sizing and purity, and sequenced in an Illumina MiSeq 250 x 250 bp paired end run. The data was demultiplexed by the MiSeq, read pairs were joined, and the resulting.fastq files for each amplicon were analyzed for indel frequencies at the guideRNA on-and off-target sites using CRISP.py (45). Lack of Elongin A expression was confirmed by immunoblotting (Fig. S2)

Eighteen to twenty-four hours before performing imaging and ChIP experiments, 3617 cells and derivatives were transferred to fresh phenol-red free DMEM supplemented with 5% Glutamax and 10% charcoal-stripped One Shot Fetal Bovine Serum without tetracycline. On the day of the experiment, GFP-GR was activated using either 100 nM of the synthetic hormone dexamethasone or natural hormone corticosterone as indicated in the figures.

### Plasmids

Plasmids encoding Halo-tagged versions of wild-type and mutant rat Elongin A (GenBank accession number AAA82095), mCherry-tagged CUL5 (mCherry-CUL5), and human Elongins B and C, and wild-type and GFP-tagged CSB have been described (10, 11).

### Chromatin immunoprecipitation and qPCR

For gene expression studies, 3617 cells were plated as described above, in 15-cm dishes 24 h before the experiment. Cells were preincubated with triptolide, α-amanitin, or vehicle for 5 min before addition of either 100 nM corticosterone or dexamethasone for the times indicated in the figures. For corticosterone wash-out experiments, corticosterone was removed by washing cells once with an excess of hormone-free medium, followed by addition of fresh hormone-free media and further incubation for up to 30 min. As indicated in the figures, cells were collected either before hormone treatment or after each treatment and harvested for further analysis.

Cells from one 15-cm dish (∼1 × 10^7^) of 3617 cells grown to 80% confluence were used for immunoprecipitation. The cells were cross-linked with 1% formaldehyde in phosphate-buffered saline (PBS) for 20 min at room temperature before quenching with glycine to a final concentration of 0.125 M, washed twice with ice-cold PBS, and resuspended in lysis buffer (15 mM HEPES, pH 7.5, 140 mM NaCl, 1 mM EDTA, 0.5 mM EGTA, 1% Triton X-100, 0.1% NaDOC, 1% SDS, 0.5% N-lauroylsarcosine, 1 mM dithiothreitol (DTT), and 1:100 Protease inhibitor cocktail (Sigma)). Cells were sonicated using a Misonix 3000 sonicator at 4 °C using output 2.5 (9 w power) for ten cycles (10 s ON/60 s OFF) to generate DNA fragments of ∼150–500 bp. Sonicated chromatin was incubated at 4 °C overnight with 5–10 μg of normal IgG or specific antibodies. The specific antibodies used were as follows: Rpb1 (D8L4Y, 14958S; Cell Signaling Technology); Elongin A (A-5, sc-373811; Santa Cruz Biotechnology); CSB (A301-345A; Bethyl Laboratories); and CUL5 custom polyclonal antibody (region 101-400aa; GenScript). Then, Dynabeads Protein A for Immunoprecipitation (10001D; Thermo Fisher Scientific) was added and incubated for 2 h at 4 °C. The beads were washed two times with IP buffer (20 mM Tris-HCl, pH 8.0, 150 mM NaCl, 2 mM EDTA, 1% Triton X-100), two times with high-salt buffer (20 mM Tris-HCl, pH 8.0, 500 mM NaCl, 2 mM EDTA, 1% Triton X-100), once with LiCl buffer (250 mM LiCl, 20 mM Tris-HCl, pH 8.0, 1 mM EDTA, 1% Triton X-100, 0.1% NP40, and 0.5% NaDOC), and two times with TE buffer. Bound complexes were eluted from the beads with 100 mM NaHCO3 and 1% SDS by incubation at 50 °C for 30 min with occasional vortexing. Cross-linking was reversed by overnight incubation at 65 °C. Immunoprecipitated DNA and input DNA were treated with RNase A and proteinase K by incubation at 45 °C. DNA was purified using the QIAquick PCR Purification Kit (28,106; Qiagen) or MinElute PCR Purification Kit (28,006; Qiagen). Immunoprecipitated and input material was analyzed by qPCR using a MyiQ Single-Color Real-Time PCR Detection System. Primer sets used for each experiment are detailed in Table S1. ChIP/Input values were calculated using the MyiQ Optical System Software. Three biological replicates were performed for each experiment.

### Microirradiation, live imaging, AP-FRET, and image analysis

Time lapse movies, UV laser microirradiation, and acceptor photobleaching–FRET (AP-FRET) measurements in individual living cells were performed essentially as described (10, 11, 46) using a PerkinElmer Life Sciences Ultra VIEW VoX spinning disk microscope with a Yokagawa CSU-X11 spinning disk, an ORCA-R2 camera (Hamamatsu), PerkinElmer Life Sciences PhotoKinesis accessory on a Carl Zeiss Axiovert 200M base equipped with a x40, 1.3 numerical aperture plan-apochromat objective. Prior to microirradiation and/or AP-FRET, CS1AN, U2OS, or 3617 cells were plated at 50–60% confluence in MatTek Glass bottom dishes (35 mm, No. 2 14 mm diameter glass) and transfected using FuGENE HD and plasmids encoding Halo-Elongin A (100 ng as donor, 400 ng as acceptor), mCherry-CUL5 (100 ng or 400 ng), and GFP-CSB (100 ng) as indicated, together with plasmids encoding Elongin B (100 ng) and Elongin C (100 ng). To label Halo-tagged proteins with rhodamine 110 or TMRDirect in living cells, media was changed after 24 h, HaloTag R110Direct (when Elongin A was the FRET donor) or TMRDirect (when Elongin A was the FRET acceptor) ligand was added to a final concentration of 100 nM, and cells were allowed to incubate overnight without washing as directed in the manufacturer's protocol. Cells were stained for 30 min with Hoechst dye to mark nuclei and/or sensitize cells to UV irradiation 24 h post transfection and allowed to recover for 5 min before AP-FRET measurements.

Values for normalized recruitment after microirradiation (*Rt*) were calculated using the equation *R* (*t*) = [*I* (*t*)/*T* (*t*)]/[*I* (*0*)/*T* (*0*)]. *I* (*0*) and *T* (*0*) are the average fluorescence intensities of the microirradiated and total nuclear region, respectively, averaged over the preirradiation time period. *I* (*t*) is the fluorescence intensity of the microirradiated stripe as a function of time and was measured as the average intensity of a manually selected region corresponding to the visible bleached region immediately after microirradiation. *T* (*t*) (total nuclear fluorescence intensity) was measured in the same way selecting the nuclear boundary. For measurements of nuclear AP-FRET, a sequence of at least three images of each nucleus was collected before and after photobleaching of the mCherry or TMRDirect photoacceptor with 15 iterations of 100% 561 nm laser power. FRET efficiencies (*E*) were calculated using the equation $E=1-I_{before}/I_{after}$ , where the brackets represent a temporal average, and *I*_*before*_ and *I*_*after*_ refer to the donor fluorescence intensity before and after acceptor photobleaching. Statistical analyses were performed in GraphPad Prism 6.

## Data availability

All data is presented in the figures and supporting information for this paper. Original data underlying this paper can be accessed from the Stowers Original Data Repository at http://www.stowers.org/research/publications/LIBP-1521.

## Supporting information

This article contains supporting information (24).

## Conflict of interest

The authors declare that they have no conflicts of interest with the contents of this article.
