# Supplementary material for: A role for the Cockayne Syndrome B (CSB)-Elongin ubiquitin ligase complex in signal-dependent RNA polymerase II transcription
Source: J Biol Chem. 2021 Jun 9;297(1):100862. doi: 10.1016/j.jbc.2021.100862 (PMC8294581; doi:10.1016/j.jbc.2021.100862)
Supplement: Table S1 and Figures S1–S4 [file mmc1.pdf]

Table S1

Gene	Amplicon Location*	Primer Name	Chromosome	Start	End	Sequence	Used in indicated ChIPs
MT1 NM_013602							
	GRE/PRO (TSS)*	GRE_MT1_FWD	chr8	94178992	94179015	TAGGGACATGATGTTCCACACGTC	GFP (GR), RNAPII, ELOA, CUL5, CSB
		GRE_MT1_REV	chr8	94179088	94179107	TTTTCGGGCGGAGTGCAGAG	
	GB (+750)	MT1_GB_FWD	chr8	94179793	94179813	CCTCATGCTGTCTTCTTTCT	GFP (GR), RNAPII, ELOA, CUL5, CSB
		MT1_GB_REV	chr8	94179882	94179902	GTGTCCCAACTCACTCTTCT	
MT2 NM_008630							
	GRE (-1.1 kb)*	GRE_MT2_FWD	chr8	94171394	94171415	CATAGCCAGGGCAGCCACAGAA	GFP (GR)
		GRE_MT2_REV	chr8	94171489	94171512	GGCAATGCCTTCTTGACTCATTCC	
	PRO-PROX (+200)	MT2_PRO_FWD	chr8	94172728	94172745	TGCGCTCGACCCAATAC	RNAPII, ELOA, CUL5, CSB
		MT2_PRO_REV	chr8	94172799	94172820	GACGAGAGATCGGTTTGAAGA	
	GB (+740)	MT2_GB_FWD	chr8	94173306	94173325	GCTCACGTTCAACTCTTCT	RNAPII, ELOA, CUL5, CSB
		MT2_GB_REV	chr8	94173391	94173410	ACTTGTCGGAAGCCTCTTT	
LCN2 NM_008491							
	GRE/PRO (-500)	LCN2_PRO_FWD	chr2	32388097	32388118	CACTATCCTGTTTCTGACCCT	GFP (GR), RNAPII, ELOA, CUL5, CSB
		LCN2_PRO_REV	chr2	32388180	32388200	TAAGCCAAAGCCATCTGAAC	
	GB (+2.9 kb)	LCN2_GB_FWD	chr2	32384724	32384744	CTGTCTGCCACTCCATCTT	
		LCN2 GB_REV	chr2	32384784	32384803	AAGATGGAGTGGCAGACAG	
TGM2 NM_009373							
	GRE/PRO (TSS)**	TGM2_GRE-PRO_FWD	chr2	158146328	158146344	GCAGCGGGACACTCAAG	GFP (GR), RNAPII, ELOA, CUL5, CSB
		TGM2_GRE-PRO_REV	chr2	158146387	158146405	GAACTTATAGCCCAGGGCG	
	GB (+28 kb)	TGM2_GB_FWD	chr2	158117874	158117893	AGCTTGTGGAGGCCAATATC	GFP (GR), RNAPII, ELOA, CUL5, CSB
		TGM2_GB_REV	chr2	158117964	158117985	AGAAGGTAGGTAAGGAGACAGG	
MMTV LTR							
	GRE/PRO (NucB)*	NucB_FWD				TGGTTACAAACTGTTCTTAAAACGAGGATG	GFP (GR), RNAPII, ELOA, CUL5, CSB
		NucB_REV				CTCAGATCAGAACCTTTGATACCAAACC	

* Approximate position relative to annotated gene TSS in mm10

** These primer sequences from:
Ultradian hormone stimulation induces glucocorticoid receptor-mediated pulses of gene transcription
Diana A. Stavreva, Malgorzata Wiench, Sam John, Becky L. Conway-Campbell, Mervyn A. McKenna, John R. Pooley, Thomas A. Johnson, Ty C. Voss, Stafford L. Lightman, Gordon L. Hager,
Nat Cell Biol. 2009 Sep; 11(9): 1093–1102. Published online 2009 Aug 16. doi: 10.1038/ncb1922

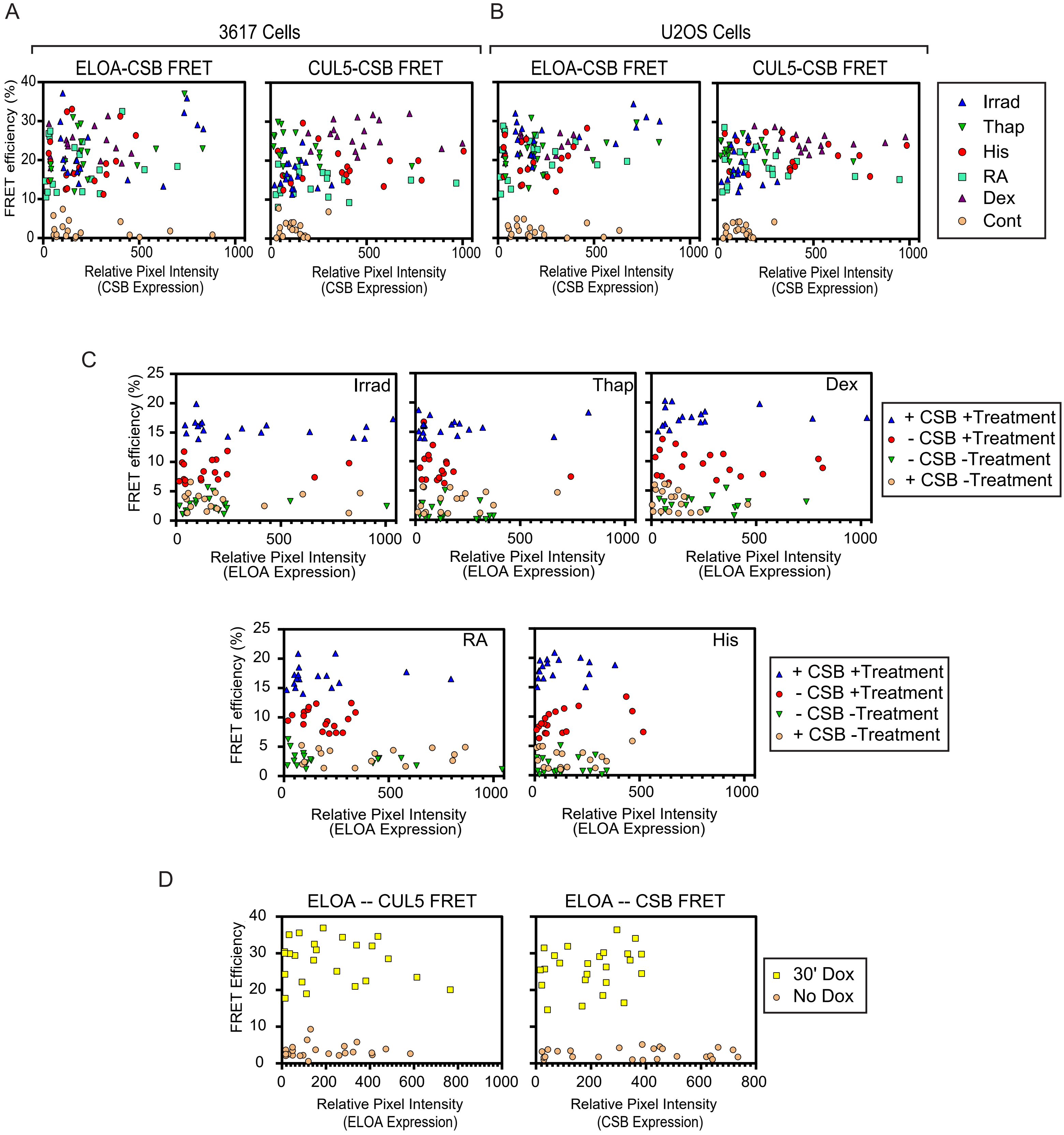

Fig. S1. AP-FRET signals are independent of donor expression levels. Scatter plots showing relative pixel intensity from donor in AP-FRET experiments, measured before acceptor photobleaching, as a function of FRET efficiency in individual cells. Panels A-D show data from the same cells used for AP-FRET experiments in Fig. 1, panels A-D, respectively. Panel D includes data from no doxycycline (Dox) and 30’ doxycycline treatment only. Irrad., microirradiation; Thap, thapsigargin; His, histidinol; RA, retinoic acid; Dex, dexamethasone; Cont, control.

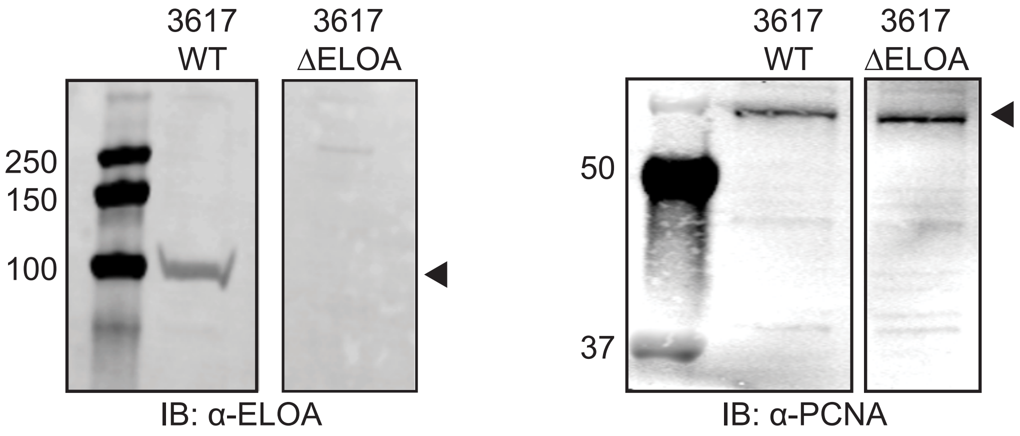

Figure S2. ELOA is not detected by immunoblotting in 3617 ΔELOA cells. Lysates from equivalent amounts of 3617 and 3617 ΔELOA cells were analyzed by immunoblotting using anti-ELOA antibodies or, as a loading control, with anti-PCNA antibodies.

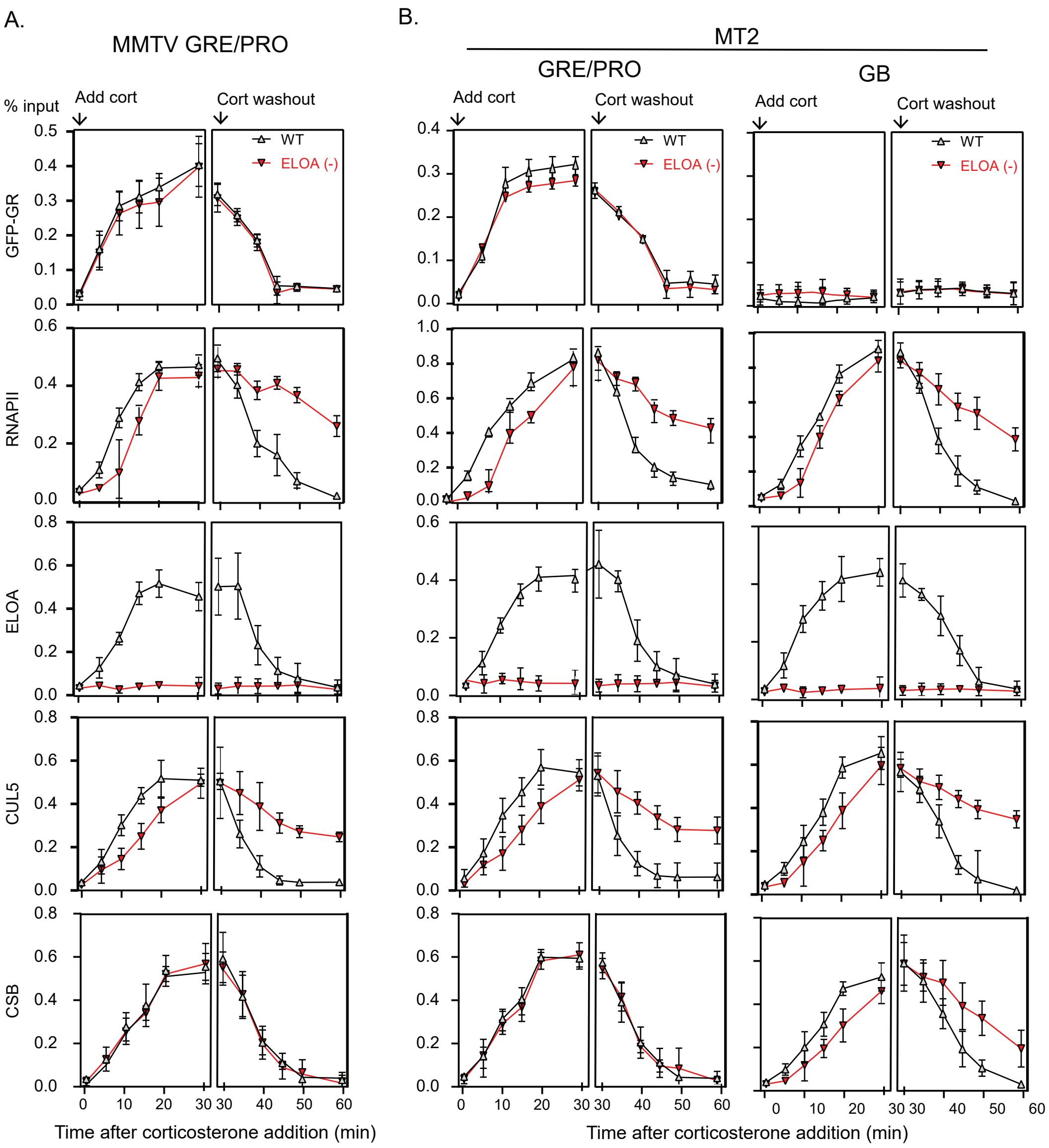

Figure S3. Altered RNAPII, CUL5, and CSB dynamics at MMTV and MT2 genes in 3617 cells lacking ELOA. Wild type or ΔELOA 3617 cells were either incubated with 100 nM corticosterone for up to 30 min, or, in corticosterone washout experiments, were incubated with corticosterone for 30 min and then washed to remove corticosterone and incubated with hormone-free medium for up to 30 minutes. Crosslinked chromatin from these cells was subjected to ChIP with antibodies against the indicated proteins. Cort, corticosterone; GRE/PRO, glucocorticoid response element and promoter region; GB, gene body.

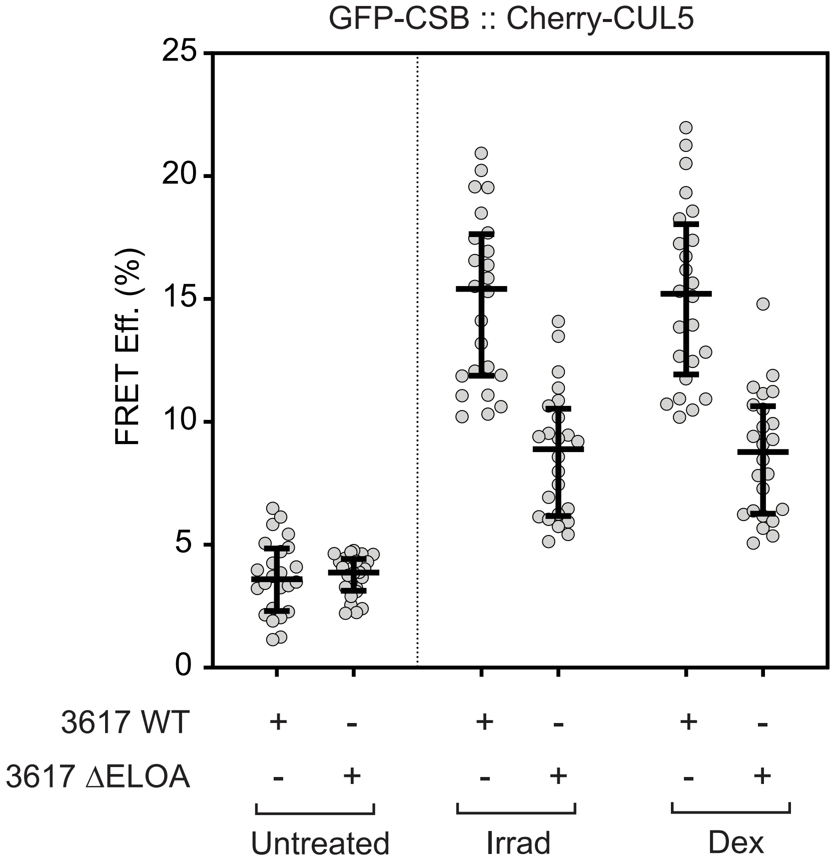

Figure S4. Loss of ELOA reduces, but does not eliminate, interaction between CSB and CUL5. AP-FRET efficiency (FRET Eff.) was measured in 3617 or 3617 ΔELOA cells transiently expressing GFP-CSB and mCherry CUL5 and subjected to either laser microirradiation (Microirrad) or treatment with dexamethasone (DEX).
